# Low-cost large-area 100 GHz intelligent reflective surface: electrically column control of screen-printable high phase changing ratio vanadium dioxides

**DOI:** 10.1515/nanoph-2025-0006

**Published:** 2025-04-15

**Authors:** Eiyong Park, Junghyeon Kim, Minjae Lee, Ratanak Phon, Mihyun Kim, Sunghoon Hong, Sungjoon Lim

**Affiliations:** School of Electrical and Electronics Engineering, 26729 Chung-Ang University, 84 Heukseok-Ro, Dongjak-Gu, Seoul, Republic of Korea; Department of Intelligent Semiconductor Engineering, Chung-Ang University, 84 Heukseok-Ro, Dongjak-Gu, Seoul, Republic of Korea; Department of Electrical and Computer Engineering, Stevens Institute of Technology, Hoboken, NJ 07030, USA; ICT Materials & Components Research Laboratory, Electronics and Telecommunications Research Institute (ETRI), 218 Gajeong-Ro, Yuseong-Gu, Daejeon, 34129, Republic of Korea; Department of Intelligent Semiconductor Engineering, School of Electrical and Electronics Engineering, Chung-Ang University, 84 Heukseok-Ro, Dongjak-Gu, Seoul, Republic of Korea

**Keywords:** VO_2_, sub-THz, IRS, high phase changing ratio, screen-printing

## Abstract

In this paper, we propose for the first time 100 GHz intelligent reflective surface (IRS) using screen-printable, high phase changing ratio vanadium dioxide (VO_2_). Sub-THz communications offer advantages such as ultra-high speed and ultra-low latency, it increases communication challenges due to path losses and non-line-of-sight (NLOS) problems. IRS is a representative solution to this NLOS problem. Conventional IRSs using PIN and varactor diodes have difficulty covering the sub-THz band due to the operating frequency limitations of these tuning elements. In this study, we successfully applied screen-printed VO_2_ switches to sub-THz IRS to control the reflection angle. The screen-printed VO_2_ achieved the highest reported available phase-changing-ratio (PCR) of 1,000, increasing design freedom. The measurement results are consistent with the numerically calculated values and EM simulation results. We believe that this solution will open new avenues and potential for practical applications in low-cost, large-area tunable RF electronics in the sub-THz band.

## 1 Introduction

Fifth-generation (5G) and sixth-generation (6G) wireless communication using millimeter wave (mm-wave) and sub-THz are expected to expand radio frequency (RF) electronics applications dramatically based on their ultra-speed, ultra-low latency, and ultra-connectivity [1], [2], [3], [4], [5]. These applications include virtual reality, augmented reality, holograms, the Internet of Things, autonomous driving, robotics, Bio RF electronics, and wearable RF electronics. However, several problems can occur in 5G and 6G, despite the advantages. First, increasing the operating frequency increases the path losses. Second, increasing the operating frequency also increases the straightness of the wave, causing communication disability in non-line of sight (NLOS) areas. To solve those problems, many researchers have proposed new scenarios and key technologies, such as low earth orbit satellite communication [6], [7], drone communication [8], [9], beam-forming antenna arrays [10], [11], and intelligent reflective surfaces (IRSs). Especially, IRSs are considered a key technology of NLOS solutions in 5G and 6G communication because they can control the electromagnetic (EM) wave path beyond Snell’s law [12], [13], [14], [15], [16], [17], [18], [19].

An IRS mainly consists of three parts: a large array of passive scattering elements, RF switches, and an embedded controller. The large array of passive scattering elements is based on a metasurface and determines the initial path of the EM wave. The RF switches change the array composition of the unit structures and determine new reflection angles. The embedded control part operates the RF switches to adapt to environmental changes by injecting coded DC signals. This alters the RF switch states, reconfigures the passive scattering elements, and forms a new EM wave path accordingly. The IRS is expected to provide a low-cost solution for indoor communication compared with other solutions (such as relays and repeaters) because it does not require an amplifying system when generating the new EM wave path. For the same reason, and in contrast, relays or repeaters using a conventional phased array antenna system when outdoors have an advantage in terms of power amplification, because the IRS cannot amplify the RF signal. As a result, the efficiency of an IRS in terms of RF performance and cost is a critical indicator of IRSs, although achieving efficiency at mm-wave and sub-THz remains a challenge for IRSs.

The unit structure of the IRS, which is part of a metasurface, is typically designed to be a quarter- or half-wavelength, similar to other metasurface unit structures [20], [21], [22]. As the operating frequency increases, the wavelength decreases, resulting in a smaller unit structure. RF switches require higher fabrication resolution because they must be placed in tight spacing where the electric field is concentrated. In addition, additional bonding materials such as solder or conductive epoxy are required to bond RF switches. However, these adhesive materials act as parasitic inductance at higher frequencies, such as sub-THz, which adversely affects stable IRS behavior. In terms of RF efficiency, the shaded area coverage effect of the IRS increases as its physical size increases [23], [24], [25], [26], [27]. To maintain the size of the IRS as the frequency increases, more unit structures and RF switches are required. Furthermore, the higher the frequency, the higher the cost of a single RF switch. As a result, at mm-wave and sub-THz, it is challenging to construct a low-cost IRS with high RF efficiency. In this study, we synthesized vanadium dioxide (VO_2_) into screen-printable VO_2_ ink with low cost, high fabrication scalability, high fabrication resolution, and tunable performance. We then apply this ink to an IRS at 100 GHz for the first time and verify the performance in a far-field environment.

VO_2_ is a smart material that changes its material properties from being a dielectric to a conductor before and after the phase-changing temperature. Accordingly, it has been deemed the best candidate for a tunable material in the THz band due to its fast transition speed and high PCR properties in the THz band [28], [29], [30], [31], [32], [33]. However, since VO_2_ is basically fabricated at high temperatures (over 400 °C) and high pressures under vacuum conditions, its application has been limited due to its small fabrication area and high cost. Recently, methods such as magnetron sputtering [34], [35], electrochemical [36], [37], and hydrothermal synthesis [38], [39], [40] have been studied to expand the application field by solving the inherent manufacturing problems of VO_2_ [41], [42]. Hydrothermal synthesis-based VO_2_ is one of the various solutions, which provides low-cost and large-area fabrication characteristics. In particular, when hydrothermal synthesis-based VO_2_ is combined with printing technologies (such as inkjet or screen printing), it can be patterned based on the high fabrication resolution of the printing technology, making it possible to apply VO_2_ to RF devices with high fabrication requirements [43], [44], [45], [46]. Nevertheless, VO_2_ has been limited in terms of application to RF devices due to its low PCR (100–300). When the PCR is low, the resistance in the ON state (acting as a conductor) could be too high or the resistance in the OFF state (acting as a dielectric material) could be too low. The resistance created in both cases would be a loss in the RF element. As a result, VO_2_ with low PCR is suitable for RF devices that require losses, such as absorbers, but is less suitable for RF devices that require low losses, such as antennas or IRS.

In this work, we propose a low-cost, large-area 100 GHz IRS utilizing screen-printable VO_2_ ink with a PCR of >1,000. To minimize the cost of IRS fabrication, the essential RF switch in IRS design was implemented using a VO_2_ switch instead of a PIN or varactor diode. The high PCR VO_2_ switch we developed increases the efficiency of the IRS. For example, the unit structure of our proposed IRS has a reflection coefficient close to −1 dB in the ON and OFF states. We fabricated a large-area 100 GHz IRS consisting of 50 rows and 50 columns using screen printing. The fabricated IRS, including the DC bias lines and pads, has dimensions of 28.6 λ_0_ × 17.8 λ_0_. The IRS controls the reflection angle through column control. The column in the ON state is ‘1’ and the column in the OFF state is ‘0’. The reflection angle control capability of the IRS was confirmed by measurements of 0000, 0101, 0011, 000111, and 00001111 under far-field conditions using a sub-THz horn antenna.

## 2 Results and discussion

### 2.1 Proposed sub-THz IRS design

**Figure 1:**
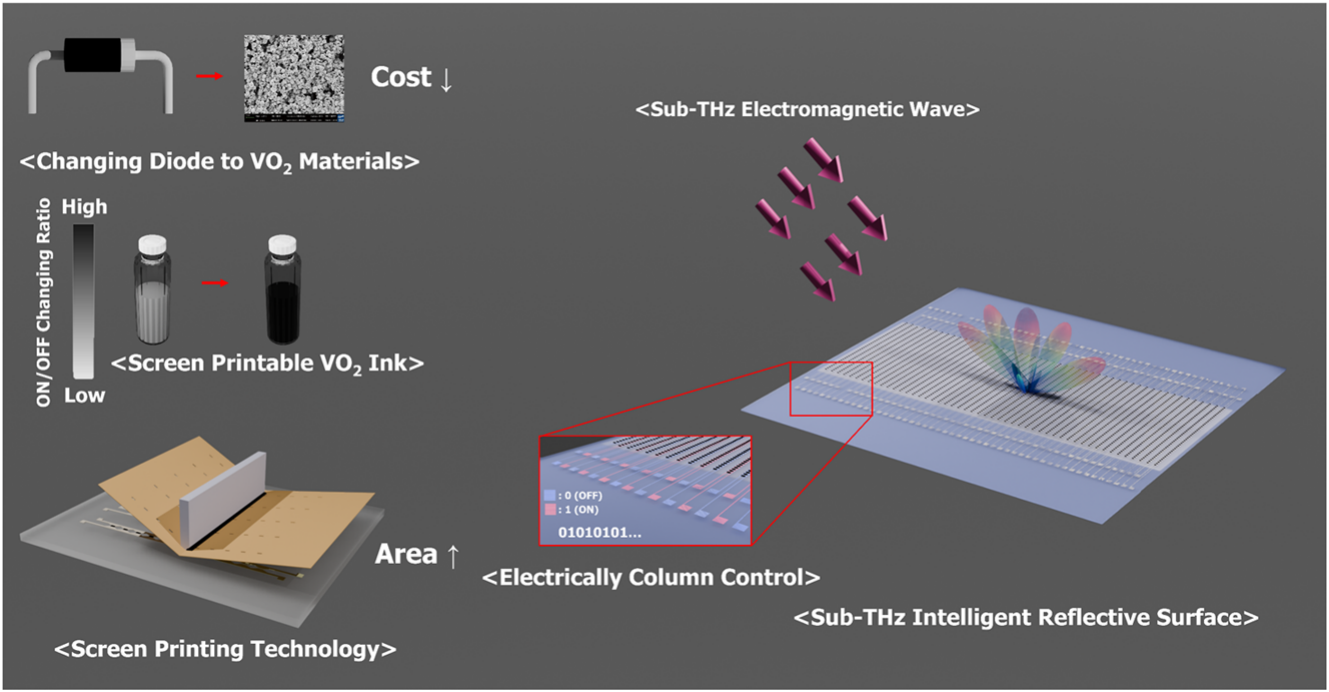
Concept illustration of the proposed sub-THz intelligent reflective surface using screen-printable high phase changing ratio VO_2_.

Figure 1 illustrates the concept of the proposed sub-THz IRS. The IRS utilizes tuning elements such as PIN diodes and varactor diodes. As the operating frequency increases, the wavelength decreases, leading to a reduction in the size of the unit structure. To create a large-area IRS, numerous tuning elements are required, which drives up costs. In this study, by using VO_2_ material instead of the commonly used tuning elements, we were able to reduce the cost, which typically rises with increasing operating frequency. At higher frequencies, parasitic inductance and capacitance caused by fine conductor patterns or gaps can easily occur. In particular, RF switches composed of patterned VO_2_ operate as conductors in the ON state, where the resulting current density induces inductance. In the OFF state, the spacing between the two conductive patterns leads to the formation of capacitance. If the OFF-state resistance of the VO_2_ switch is low, it does not function as a perfect insulator, causing unintended inductance to form in the OFF state. This undesired inductance degrades the efficiency of the IRS. Therefore, the resistance in the OFF state must be high, which requires a VO_2_ switch with a high PCR. We enhanced the PCR of VO_2_ switches, which typically have a PCR of 300 or less when fabricated using conventional hydrothermal synthesis, to over 1,000, making them suitable for sub-THz IRS applications. Additionally, we developed the synthesized VO_2_ material into a screen-printable ink, facilitating large-area sub-THz IRS fabrication. Our proposed IRS employs electrically column control, enabling simple beam steering of reflected waves, with a straightforward control circuit.

Beam steering using VO_2_ column control can be realized by controlling the array factor (AF), as follows:

(1) $$\left|AF\left(\theta \right)\right|=\left|\overset{2N}{\underset{n=1}{\sum }}e^{-j\left(kD\left(n-1\right)\sin\theta {+\emptyset }_{n}\right)}\right|\cdot \left|1+e^{-j\left(KDsin\theta -\Delta \emptyset \right)}\right|.$$

Here, *k* is the wave number, *D* is the distance between unit structures, and *ϕ* is the phase. The reflective beam is formed at *θ*, where AF is maximized according to the following equation:

(2) kDsinθ−Δϕ=γ2π,

where *γ* is the order of the grating lobe in an array antenna. When the phase difference of the unit structure is 180°, Equation (2) becomes the following:

(3) $$\sin\theta_{r}=\left(2\gamma +1\right)/2\cdot \lambda /D$$

As a result, in the fundamental mode, *γ* in Equation (3) becomes zero, and the reflection angle is determined solely by the spacing *D* between unit structures with the same bit. For example, at 100 GHz, if the spacing *D* between one ON-state unit structure and the next ON-state unit structure is 1.7 mm, the reflection angle is 61.9°. Similarly, at 100 GHz, if the spacing is 3.4 mm, the reflection angle is 26.2°. To control the reflected waves, two conditions are required in the unit structure. The first is that the reflection coefficient of the unit structure must be higher than −3 dB. As the reflection coefficient of the unit structure decreases, the intensity of the reflected electromagnetic wave also decreases, leading to reduced efficiency of the IRS. The second is that there must be a 180° phase difference between the ON and OFF states of VO_2_.

**Figure 2:**
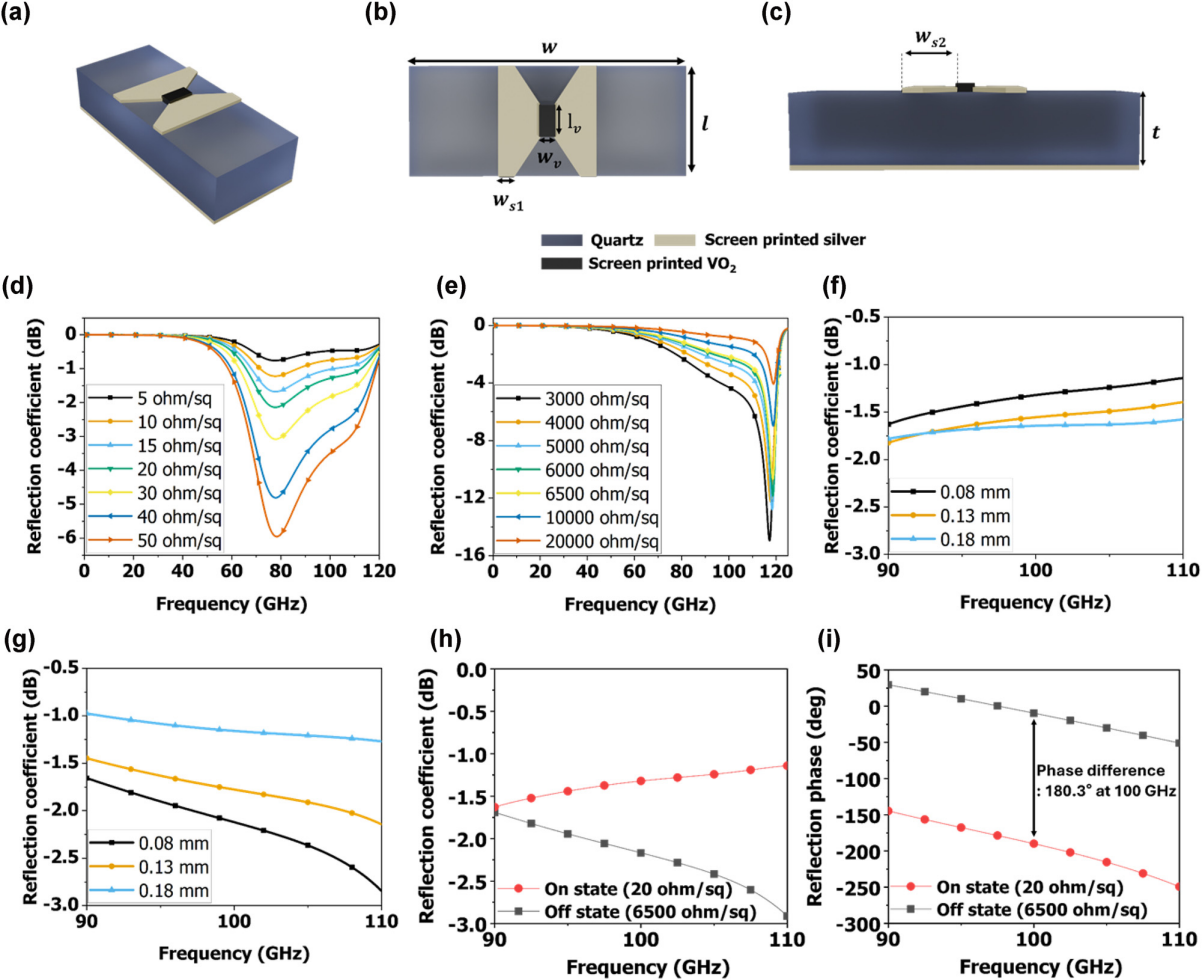
Unit structure (a) birds-eye view, (b) top view, (c) side view of the proposed IRS (*w* = 1.7 mm, *l* = 0.7 mm, *w*_*v*_ = 0.1 mm, *l*_*v*_ = 0.2 mm, *w*_*s*1_ = 0.1 mm, *w*_*s*2_ = 0.26 mm, and *t* = 0.35 mm). Simulation results of the unit structure. Simulation results of the unit structure. (d) Reflection coefficient when VO_2_ sheet resistance is from 5 to 50 Ω/sq. (e) Reflection coefficient when VO_2_ sheet resistance is from 3,000 to 20,000 Ω/sq. Simulated reflection coefficient results for various *w*_*v*_ values when the sheet resistance of the VO_2_ switch is (f) 20 Ω/sq, and (g) 6,500 Ω/sq. (i) Simulated reflection phase when VO_2_ sheet resistance are 20 and 6,500 Ω/sq.

Figure 2(a–c) shows birds-eye, top, and side views of a unit structure of the proposed IRS, respectively. We selected a geometry that narrows from the outside to the inside to maximize the thermal effect applied to VO_2_ by structurally reducing the width of the current-carrying lines. We designed a unit structure that integrates both a metasurface pattern and a biasing line while minimizing heat dispersion. VO_2_ undergoes phase transitions when heated by electrical signals. To optimize performance, we adjusted the distance between the heated and room-temperature VO_2_ switches and minimized the size of the heated VO_2_ switch. The top layer consists of a screen-printed silver pattern and screen-printed VO_2_. The bottom layer consists of a screen-printed silver ground. We used 0.35-mm-thick quartz substrate with a dielectric constant of 3.8 and a loss tangent of 0.0012 at 100 GHz (Hankuk Advanced Materials, South Korea). For the silver, we used screen-printable silver ink (DM-SIP-3063S, DycoTec, UK). We employed the ANSYS high-frequency structure simulator (HFSS) tool for both the unit structure and full structure analyzes. Figure S1 shows the boundary setup used for the unit structure simulation, in which we implemented periodic boundary conditions in HFSS. Figure 2(d) shows the simulated reflection coefficient results when the VO_2_ switch in the unit structure was in the ON state. The resonant frequency occurred at 80 GHz, and the reflection coefficient decreased as the sheet resistance of the VO_2_ switch increased. When the sheet resistance of the VO_2_ switch increased from 5 to 20 Ω/sq, the reflection coefficient remained higher than −3 dB across the entire frequency range from 90 to 110 GHz. However, starting from 30 Ω/sq, the reflection coefficient in some frequency bands dropped below −3 dB, and the bandwidth with a reflection coefficient below −3 dB between 90 and 110 GHz expanded as the sheet resistance increased. In the ON state, lower VO_2_ switch resistance resulted in a higher reflection coefficient. Figure 2(e) shows the simulated reflection coefficient results when the VO_2_ switch in the unit structure was in the OFF state. As the sheet resistance of VO_2_ increased from 3,000 to 20,000 Ω/sq, the −3 dB frequency range shifted from 92 to 110 GHz. It can be seen that for the reflection coefficient to stay above −3 dB in both the 90–110 GHz bands, the VO_2_ sheet resistance must be higher than 6,500 Ω/sq. The higher the sheet resistance, the higher the reflection coefficient in the 90–110 GHz frequency range. From these results, we conclude that the unit structure of our proposed IRS is more efficient when the VO_2_ switch has a lower sheet resistance in the ON state and a higher sheet resistance in the OFF state. We simulated the effect of the VO_2_ switch placement area on performance. Figure 2(f and g) presents the simulation results of the reflection coefficient as a function of the VO_2_ switch placement area (*w*_*v*_) when the sheet resistance of the VO_2_ switch is 20 Ω/sq and 6,500 Ω/sq, respectively. In Figure 2(f), when *w*_*v*_ = 0.08 mm, the reflection coefficient was greater than −1.7 dB, and as *w*_*v*_ increased, the reflection coefficient decreased. Conversely, in Figure 2(g), when *w*_*v*_ = 0.08 mm, the reflection coefficient was lower than −3 dB, and as *w*_*v*_ increased, the reflection coefficient increased. The reason for the opposite trend is that, as the VO_2_ switch size increases, the resistance of the VO_2_ switch rises in both ON and OFF states, causing different changes in the reflection coefficient. For instance, as shown in Figure 2(d and e), when the resistance of the VO_2_ switch increases, the reflection coefficient decreases in the ON state but increases in the OFF state. Figure 2(h) shows the reflection coefficient simulation results for VO_2_ in the 90–110 GHz frequency band when the ON and OFF state sheet resistances of VO_2_ were 20 and 6,500 Ω/sq, respectively. Here, it is evident that both the ON and OFF states had a reflection coefficient higher than −3 dB in the 90–110 GHz frequency band. In the current structure, to achieve a −3 dB reflection coefficient, a PCR of at least 325 was required. To achieve a −1 dB reflection coefficient, the sheet resistance of VO_2_ was 15 Ω/sq in the ON state and 10,000 Ω/sq in the OFF state, which required a PCR of at least 670. As the PCR of the VO_2_ increases, the efficiency of the proposed IRS also increases. Figure 2(i) shows the on and off state of the reflected phase simulation results for the 90–110 GHz frequency band, showing a phase difference of 186.3° ± 12.1° within the 90–110 GHz frequency band.

**Figure 3:**
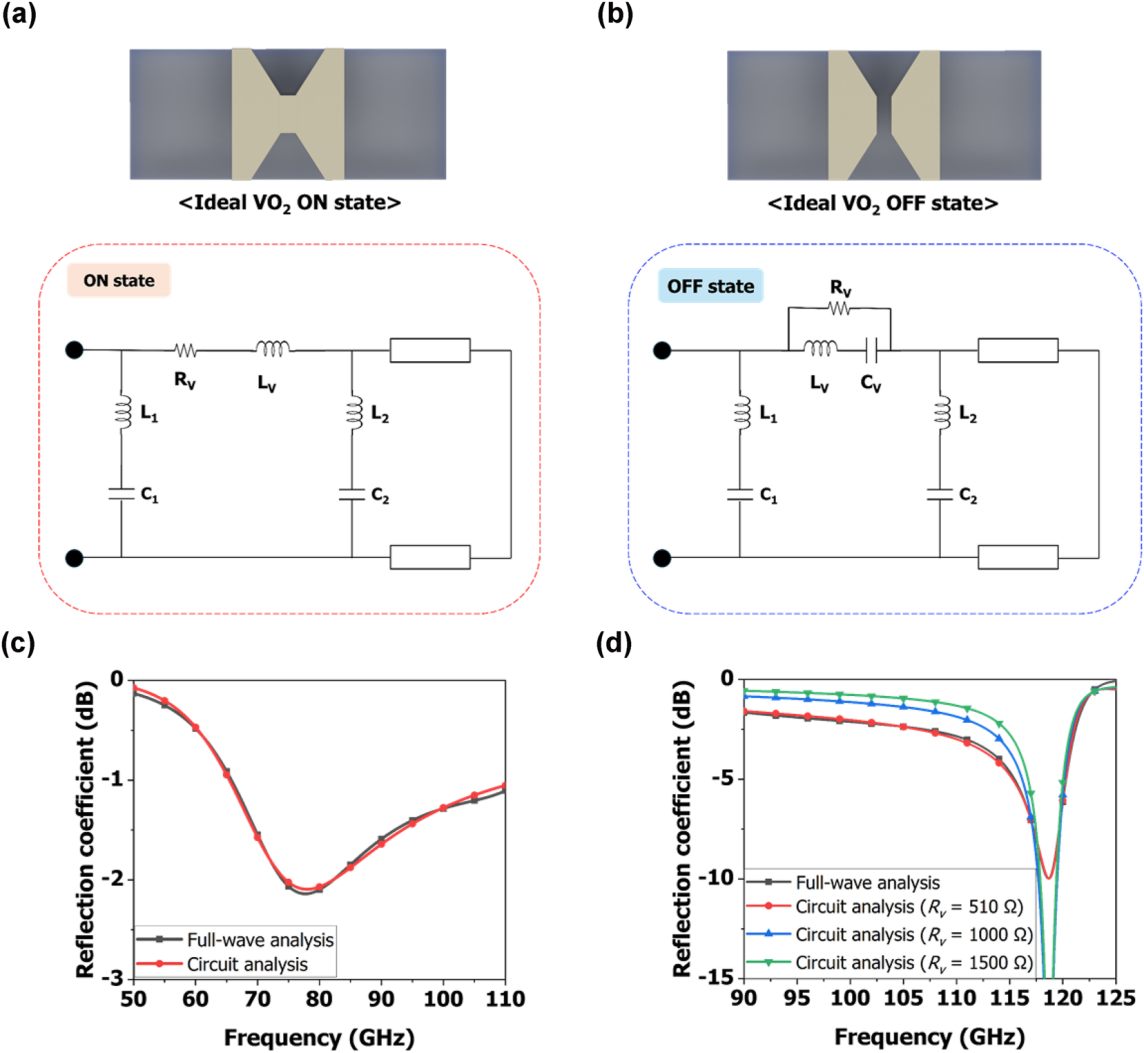
Equivalent circuit of the unit structure with the VO_2_ switch in (a) ON state and (b) OFF state. Comparison of the reflection coefficients obtained from the full-wave analysis and circuit analysis for (c) ON state, and (d) OFF state.

Figure 3(a and b) shows the equivalent circuit of a unit structure in the ON and OFF states of the VO_2_ switch. When on, the resistance and inductance of the VO_2_ switch are connected in series between two series inductance, capacitance circuits configured in parallel. When in the OFF state, the series-parallel resistance, inductance and capacitance of the VO_2_ switch are connected in series between the two series inductance, capacitance circuits. In the ideal OFF state, the inductance of VO_2_ is zero and the resistance is infinite. However, a low PCR of the VO_2_ will reduce the efficiency of reflection by having low resistance even in the OFF state. We used the Keysight advanced design system tool for circuit analysis to verify agreement with the full-wave analysis performed with the HFSS tool. Figure 3(c) presents a graph comparing the full-wave analysis result of the ON-state unit structure with the circuit analysis result, showing a consistent overall trend. The circuit parameters used for the circuit analysis of the unit structure are *L*_1_ = 0.076 nH, *L*_2_ = 1.11 fH, *C*_1_ = 0.235 pF, *C*_2_ = 0.075 pF, *R*_*v*_ = 13 Ω, and *L*_*v*_ = 0.01 nH. Figure 3(d) shows a graph comparing the full-wave analysis result of the OFF-state unit structure with the circuit analysis results. We analyzed the circuit for different values of *R*_*v*_. The full-wave analysis result was consistent when *R*_*v*_ was 510 Ω. The circuit parameters used for the circuit analysis are *L*_1_ = 0.242 nH, *L*_2_ = 0.316 nH, *C*_1_ = 50 pF, *C*_2_ = 0.01 pF, *L*_*v*_ = 4.28 nH, and *C*_*v*_ = 0.39 fF. The circuit analysis results show that the reflection coefficient in the 90–110 GHz band increases as *R*_*v*_ increases. Both the full-wave analysis and the circuit analysis indicate the need for a VO_2_ switch with a high PCR.

### 2.2 Screen-printable VO_2_ ink synthesis

**Figure 4:**
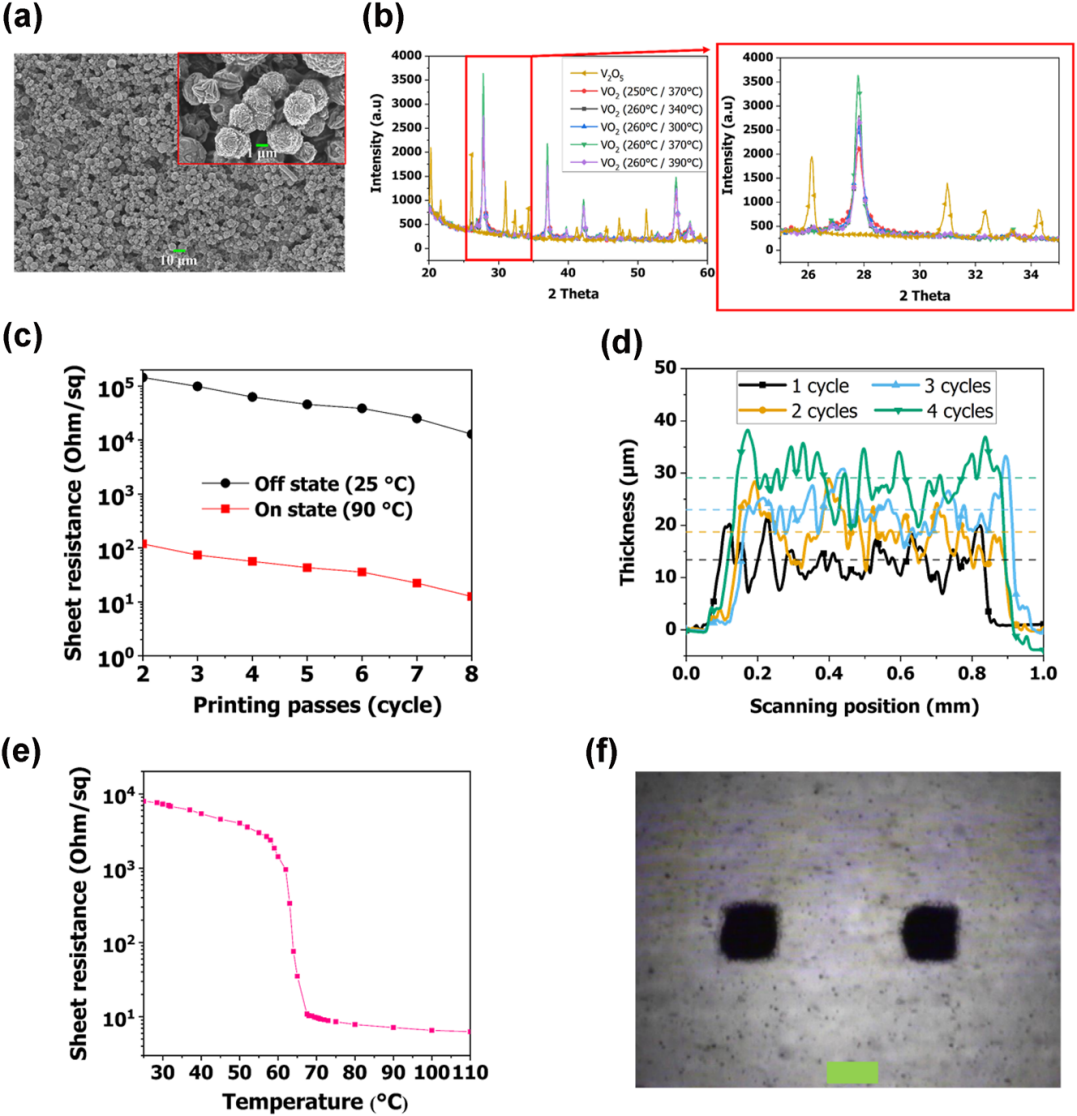
Material characterization of the screen-printed VO_2_ film. (a) SEM photograph of screen-printed VO_2_ film. (b) XRD spectra for different reaction temperatures. (c) Measured sheet resistance of the screen-printed VO_2_ switch for different printing passes. (d) Measured thickness of screen-printed VO_2_ patterns for different screen-printing cycles. (e) Sheet resistance of screen-printed VO_2_ switch for different temperatures. (f) Microscope image of a screen-printed VO_2_ patterns for 0.2 mm × 0.1 mm (green scale bar: 200 µm).

In this study, we synthesized VO_2_ microparticle (μP) with a PCR of 1,000 (or more) through hydrothermal synthesis and mixed it with an optimized ink formulation to develop a screen-printable ink (see the Materials and methods section and Figure S2). We referenced the VO_2_ synthesis and ink formulation preparation methods from a previously reported study [45]. Subsequently, we optimized the reaction time, annealing time, and temperature during the VO_2_ synthesis process and successfully synthesized VO_2_ μP with high PCR. Figure S3(a, b) shows the fabricated screen-printable VO_2_ ink. Figure S3(c, d) presents a photograph of a sample that has been screen printed with the fabricated screen-printable VO_2_ ink, where it is evident that the different patterns organized on the sample have been clearly printed. Figure 4(a) presents an SEM photograph of the as-prepared screen-printed VO_2_ film, where it is evident that the as-prepared VO_2_ formed in clusters with a rod-like morphology. Figure 4(b) shows X-ray diffraction (XRD) spectra that provide the structural and crystallinity characteristics depending on the VO_2_ μP synthesis conditions. We fixed the hydrothermal synthesis reaction time at 6 h and the annealing time of the dried VO_2_ powder at 3 h then measured the XRD patterns for different temperature conditions. XRD peaks at 27.84°, 37.01°, 42.19°, and 55.56° were indexed to the (011), (200), (210), and (−222) crystal planes, respectively, which were consistent with JCPDS No. 72–0514 [47]. During the VO_2_ synthesis process, we varied the reaction temperature between 250 °C and 260 °C, and the annealing temperature between 300 °C and 390 °C. The results showed that VO_2_ exhibited the highest crystallinity when the reaction temperature was 260 °C and the annealing temperature was 370 °C. Conversely, the lowest crystallinity was observed at a reaction temperature of 250 °C. By optimizing the annealing temperature, we can achieve high crystallinity. Figure 4(c) shows the measured sheet resistances of the screen-printed VO_2_ switches with increasing printing passes from 2 to 8 at an ON state of 25 °C and an OFF state of 90 °C. The sheet resistances in the ON and OFF states were 119 and 145,000 Ω/sq for two screen-printing cycles, respectively. Furthermore, the sheet resistances gradually decreased as the number of printing cycles increased. After eight screen-printed cycles, the sheet resistances in the ON and OFF states were measured as 12.6 and 12,900 Ω/sq, respectively, and the ON and OFF states PCR were measured at between 1,023 and 1,336 for screen-printing cycles two to eight. Figure 4(d) shows the thickness measurement results of the VO_2_ pattern for different screen-printing cycles. The thickness of the VO_2_ pattern increased by 5.5–6 µm per screen-printing cycle. Figure 4(e) presents the sheet resistance variations with temperature of the screen-printed VO_2_ switch. The VO_2_ switch sheet resistance confirmed that the VO_2_ phase changed rapidly at 64 °C. Above 80 °C, it exhibited a resistance of less than 8 Ω/sq, while below 30 °C it exhibited a resistance of over 8,000 and up to 9,400 Ω/sq. Figure 4(f) displays a microscope image of screen-printed VO_2_ patterns measuring 0.2 mm × 0.1 mm. The final size of the screen-printed VO_2_ pattern was close to 0.2 mm × 0.2 mm square. Although the printed size increased due to the spreading of the ink after screen printing, the fabricated screen-printable VO_2_ ink had a fabrication resolution that would be applicable for the proposed sub-THz IRS.

### 2.3 Proposed sub-THz IRS full structure simulation

**Figure 5:**
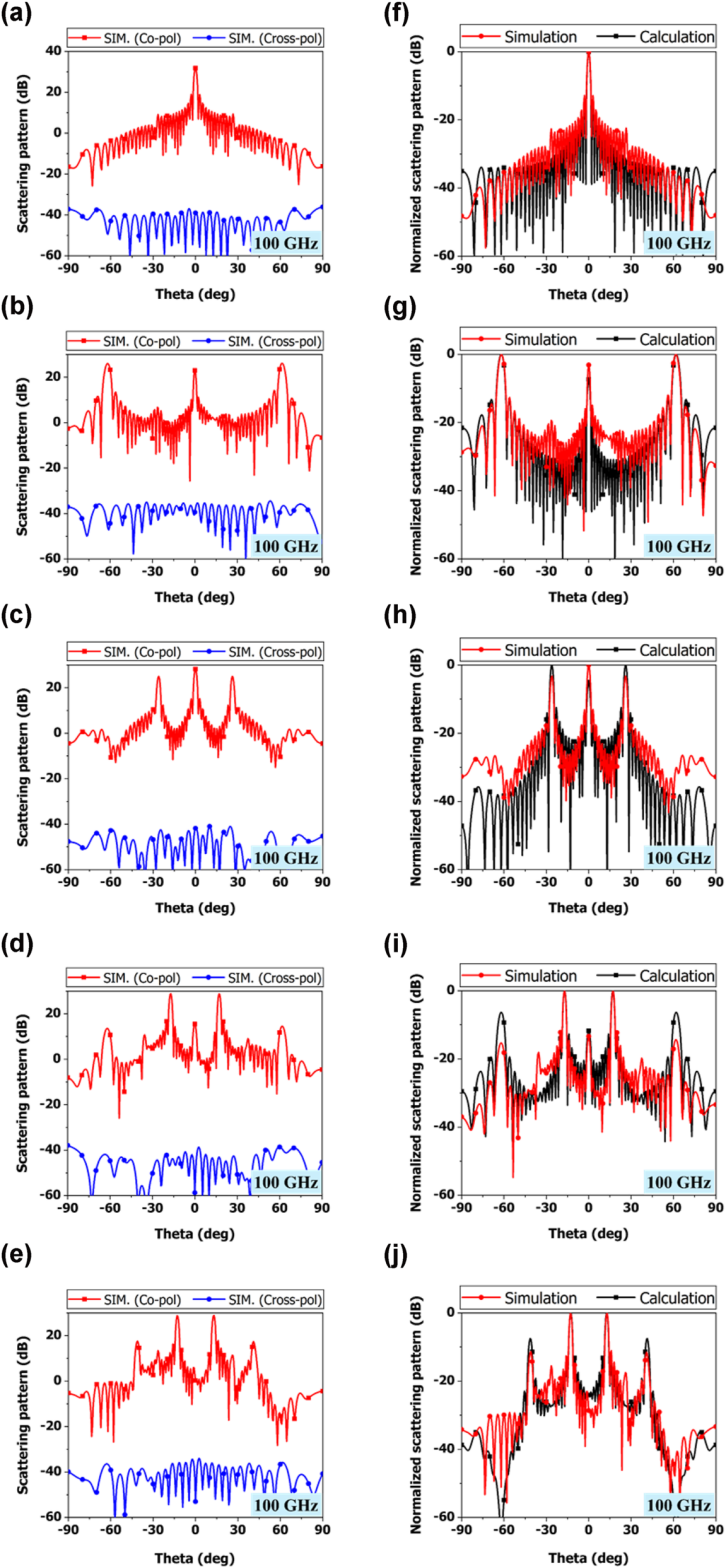
Simulation results of co-polarization and cross-polarization scattering patterns at 100 GHz: (a) 0000, (b) 0101, (c) 0011, (d) 000111, (e) 00001111. Comparison between simulation and calculation results at 100 GHz: (f) 0000, (g) 0101, (h) 0011, (i) 000111, and (j) 00001111.

We simulated the full structure of the proposed IRS using the measured sheet resistance results. We also considered the impact of align marks used during the screen-printing process and the DC biasing line in the simulation. Figure 5 shows the full structure simulation results of the final designed VO_2_ sub-THz IRS. The simulation was performed by setting different sheet resistances for each column with different coding sequences. Code 0 represented a VO_2_ sheet resistance of 10,000 Ω/sq and Code 1 represented a VO_2_ sheet resistance of 10 Ω/sq. Figure 5(a–e) shows the simulation results of the co-polarization and cross-polarization scattering pattern when the codes were 0000, 0101, 0011, 000111, and 00001111. We controlled the sub-THz IRS code through the columns. Here, code 0101 means that the states in the first through fourth columns were 0101 in sequence, and this pattern repeated until the 50th column. For example, code 0101 means 01010101…, code 000111 means 000111000111…, and so on. For code 0000, the reflected beam had an angle of 0°, because *D* is infinite. The reflected beam had an intensity of 31.8 dB, while the cross-polarized wave had a characteristic below −30 dB at all angles. For code 0101, the *D* sub-arrays were 1.7 mm apart, and the beam should be formed at ±61.9°. The simulation results showed the angle of the reflected beam to be similar to the calculated value of ±62°. The reflected wave had an intensity of 25.9 dB, and as with code 0000, the cross polarization was formed below −30 dB. For the 0011 case, the size of the sub-array was 3.4 mm, meaning the beam should be formed at ±26.2°. The simulation showed the angle of the reflected beam to be ±26°, which is similar to the calculated value. The reflected wave had an intensity of 25 dB, with the reflection at 0°, i.e. the middle beam, having a higher intensity. The cross polarization was kept at a level below −40 dB. For code 000111, the beam should be formed at ±17.1° because *D* is 5.1 mm. The simulation showed that the angle of the reflected beam was similar to the calculated value of ±17°. The reflected wave had an intensity of 28.7 dB, while the cross-polarization remained at a level below −30 dB, similar to the previous code. For code 00001111 the beam should be formed at ±12.74° because *D* is 6.8 mm. The simulation resulted in an angle of the reflected beam of ±13°, which is consistent with the calculated value. The reflected wave had an intensity of 28.8 dB, and cross-polarization was formed below −30 dB. We have simulated for five different codes so far and found that beam steering is possible for all angles except 30–60°. Of course, beam steering for angles between 30° and 60° is possible with different kinds of code arrangements. For example, Figure S4 shows the calculation results for codes 100, 01101, and 1010110. Code 100 has a reflected beam angle of ±36°, code 01101 has a reflected beam angle of ±45°, and code 1010110 has a reflected beam angle of ±49°. These results show that beam steering is possible with the column control of the various IRS code types we propose. The simulation results were the same as the expected values calculated using Eq. (3). Figure 5(f–j) presents a graph comparing the full structure simulation results with the calculated results for codes 0000, 0101, 0011, 000111, and 00001111. For all codes, the reflected beam angles matched between the simulated and calculated results, and the sidelobes exhibited similar trends. For example, in the calculated results for code 000111 in Figure 5(i), the sidelobes formed at ±62° exhibited a similar trend in the simulation results. Similarly, in the calculated results for codes 0101 and 0011 in Figure 5(g) and (h), the trend of the middle beam formed at 0° was also observed in the simulation results.

### 2.4 Fabrication and measurement

**Figure 6:**
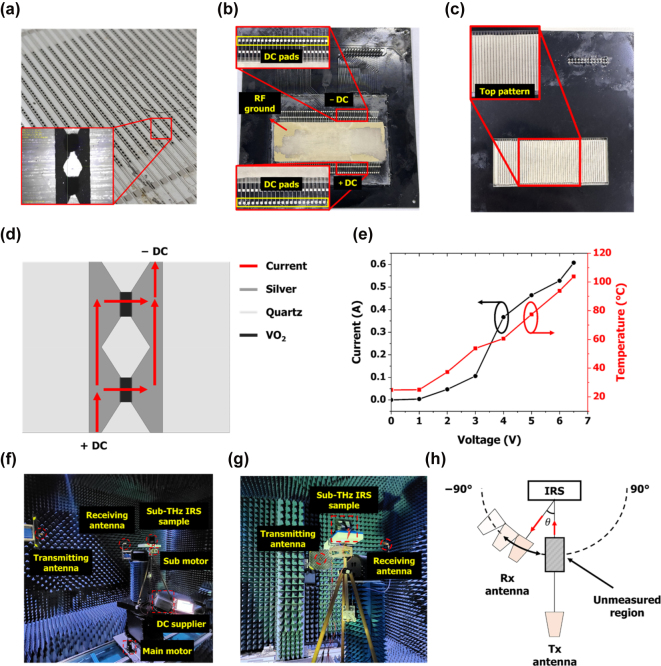
Fabrication and measurement setup of the proposed sub-THz IRS. (a) Image of fabricated sample. (b) Fabricated full structure of the IRS: (b) bottom view, (c) top view. (d) Current flow when the VO_2_ switch is ON. (e) Changes in current and temperature as the DC voltage varies in a single column of a sub-THz IRS. Measurement setup photographs: (f) bird’s-eye view, (g) back-side view, and (h) schematic illustration of the setup.

The top and bottom silver patterns and VO_2_ pattern of the IRS were created using screen printing. Figure S5 details the process of fabricating the proposed IRS. Figure 6(a) shows an image of the fabricated sample achieved by laminating 1 layer of silver and 15 layers of VO_2_. To align the silver ink and VO_2_ ink, we first inserted a cross-shaped alignment marker into the screen-printing mask. When the silver ink is printed onto the quartz substrate, both the pattern and the cross-shaped alignment markers are formed. Then, the patterned quartz substrate is removed, and the alignment markers embedded in the VO_2_ mask are printed onto the work plate. Finally, using the microscope built into the screen printer, we matched the alignment markers printed with silver and VO_2_ ink, ensuring accurate patterning of the top layer. We used DC lines and pads for column control, which were printed on top of each column. Additionally, the microscope images demonstrate that the fabricated sample was the same as the proposed design. Figure 6(b and c) shows the bottom and top views of the fabricated IRS, which was connected to the DC control system for column control. First, the connection board between the IRS and DC control system was fabricated using a PCB process. Here, a 1.6-mm-thick FR-4 substrate was used for the connection, and gold was used for patterning, which is suitable for flip-chip adhesion methods. Finally, a flip chip adhesion method was used to bond the IRS. Flip chip adhesion is a semiconductor packaging technology in which a die is flipped and directly connected to a substrate. First, under bump metallization (UBM) is formed on the chip’s pads, followed by solder bump formation through deposition or electroplating. The chip is then aligned and bonded to the substrate using reflow or thermo-compression bonding (TCB). Finally, underfill is applied to enhance mechanical strength and thermal stability, completing the process. As shown in Figure 6(b), additional DC bias lines and pads were inserted at both ends of the IRS column. Here, the +DC line is connected to the DC pad on one end, while the −DC line is connected to the other end. When voltage is applied, current flows through the unit cells functioning as DC bias lines, as depicted in Figure 6(d), and also through the VO_2_ switches. According to Joule’s law, electrical energy is converted into heat, causing the VO_2_ switches to turn ON. We measured the changes in current and temperature as the DC voltage varied in a single column of the sub-THz IRS. Figure 6(e) presents the results, showing that as the applied DC voltage to the column increased, both current and temperature also increased. In particular, when the voltage changed from 3 V to 4 V, the current exhibited a sharp increase due to the abrupt phase change of the VO_2_ switch, which significantly reduced its resistance. To reach a temperature of 100 °C, a voltage of 6.5 V was required. The reason for the 100 °C temperature is to reliably turn on all the VO_2_ switches. Figure 6(f and g) shows the birds-eye and back side views of the measurement environment, which comprises a transmitting (Tx) antenna, receiving (Rx) antenna, main rotating system, sub-rotating system, IRS, and DC power supply. Figure 6(h) shows a schematic illustration of the measurement setup. We positioned the Tx antenna and IRS so that they are facing each other, meaning that the EM waves are incident on the IRS at 0°. To measure the magnitude of the reflected EM waves, we rotated the Rx antenna using the main motor and sub motor within a range of −90° to 90°, taking measurements at 2° intervals. When the main motor rotates, both the Rx antenna and the IRS rotate simultaneously. To compensate for the IRS’s rotation, we utilized a sub-motor that controls only the IRS’s rotation angle. For example, if the main motor rotates 30°, the Rx antenna and IRS will also rotate 30°, but if we rotate the sub motor to −30°, the resulting rotation angle of the IRS will be 0°. The distance between the Tx antenna and the IRS was 2 m, the distance between the Rx antenna and the IRS was 0.8 m, and the measurements were conducted in the far-field measurements. We set different distances for the Tx antenna and Rx antenna because the sub-THz IRS was measured in a mm-wave antenna chamber, which had limited space for the Rx antenna. As a result, we could not position the Rx antenna at the same distance as the Tx antenna. From −8° to +8° was the unmeasured region where the Rx antenna overlapped the Tx antenna.

**Figure 7:**
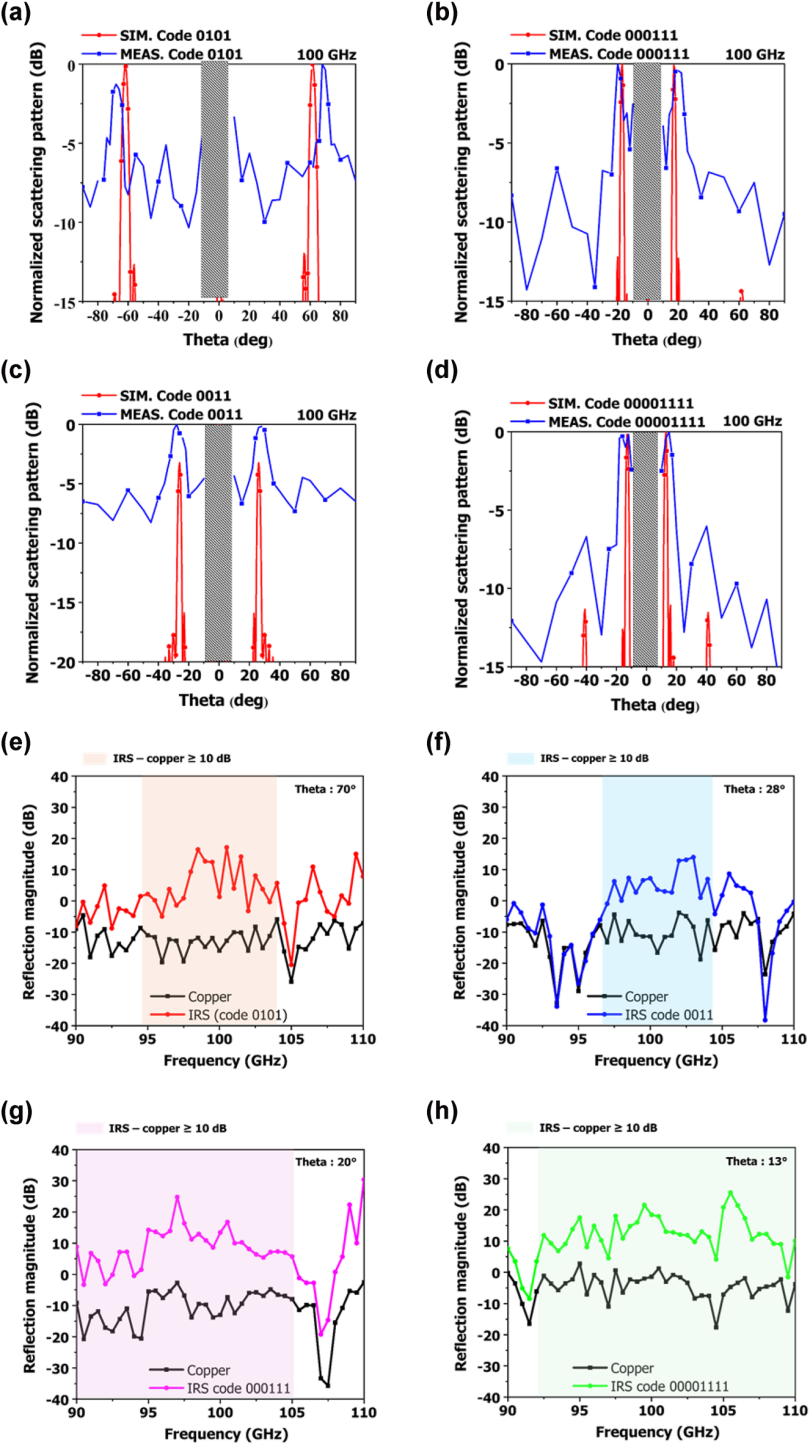
Measurement results of normalized scattering pattern for different codes: (a) 0101, (b) 0011, (c) 000111, and (d) 00001111. Measured reflection magnitude of copper and IRS (e) coding 0101 at theta 70°, (f) coding 0011 at theta 28°, (g) coding 000111 at 20°, and (h) coding 00001111 at theta 13°.

Figure 7(a–d) shows the measurement results for the different codes. The measurements were performed for codes 0101, 0011, 000111, and 0000111. The proposed IRS combines a VO_2_ switch with a DC line. When a voltage is applied to the DC line, current flows through the VO_2_ switch, and according to Joule’s law, electrical energy is converted into heat. By controlling the heat generated during this process, if the temperature exceeds 67 °C, the phase transition temperature of VO_2_, the VO_2_ switch turns on. We connected the 50 columns of the IRS to the DC supply and applied a voltage of 6.5 V to measure the angle of reflection per code. When observed with an infrared camera, we measured the 2 A current output from the DC supply when the VO_2_ switches temperature exceeded 90 °C, that is, when the VO_2_ switches were turned ON. Therefore, the total power used was 6.5 V at 2 A, and the power consumption per VO_2_ was 10.4 mW. From the measurements, it is evident that 0101 split the reflected wave at +70° and −68°. For 0011, 000111, and 00001111, the reflected waves were split at ±29°, ±20°, and ±13°, respectively. In all cases, the trends followed the simulation data. In the case of 0101, the deviation of the left and right beams was the largest because the main motor axis and the sub-motor axis were not perfectly matched in the measurement environment. Hence, when the angle changed significantly, the area where the Tx incidence hit was slightly deviated. Additionally, compared to the simulation results, a higher side lobe level was observed in the measurement results. The reason is that scattering occurred from the gold-patterned FR4 substrate used during the measurement, causing electromagnetic waves to be received not only at the designated reflection angle of the IRS, but also at other unintended angles. As shown in Figure S6(a), we implemented the actual measurement environment in the simulation. We simulated two cases: one where the IRS includes a gold-patterned FR4 substrate, and one without it. Figure S6(b) presents the simulated scattering patterns for both cases. When the IRS did not include the FR4 substrate, the side lobe was approximately 14 dB. However, when the FR4 was included, the side lobe increased to around 5 dB, and overall side lobes increased across all angles. Figure 7(e–h) shows the effect of frequency on the reflected wave formed by each code. At the reflected point, the difference in reflection magnitude between the copper and IRS is shown. For example, the measurement results for code 0101 showed the highest reflected intensity at 70°. We fixed the Tx antenna at 0° and the Rx antenna at 70°, then measured and compared the cases where an IRS and copper were placed, respectively. For other codes, the Rx antenna was changed at the angle with the highest reflected intensity, and the measurements were conducted using the same method. For 0101, the IRS had a 10 dB higher reflection magnitude compared to copper from 94.5 to 104.5 GHz. For 0011, the IRS had a 10 dB higher reflection magnitude compared to copper from 97 to 106 GHz. For 000111, the IRS had a 10 dB higher reflection magnitude compared to copper from 90 to 105.5 GHz. For 00001111, the IRS had a 10 dB higher reflection magnitude compared to copper from 92 to 110 GHz. The 0011 code, which has a wider reflection angle compared to the 000111 and 00001111 codes, had the narrowest bandwidth. The 0101 code, which has the widest reflection angle, also had a narrower bandwidth compared to other codes. All codes had a fractional bandwidth of more than 8.8 %.

Table 1 compares the performance of the proposed sub-THz IRS with previous reconfigurable metasurface studies. The authors of [48] demonstrated a reconfigurable metasurface antenna operating at 2 GHz using PIN diodes. PIN diodes have a very fast switching speed in the order of a few nanoseconds and provide stable switching performance. For example, in the ON state, they exhibit a low resistance close to 2 Ω, while in the OFF state, they have a high resistance exceeding hundreds of ohm. However, as the operating frequency increases, the cost rises significantly, making their application challenging in high-frequency bands such as 100 GHz. Additionally, as the number of unit cells increases, the manufacturing cost rises sharply, posing a significant drawback. The authors of [49], [50] proposed an RIS utilizing liquid crystal (LC). Previous studies have validated its performance in high-frequency bands, including 100 GHz, and demonstrated large-area fabrication at a low production cost. However, it has a slow phase transition speed in the range of ms to sec, and its multilayer design requirement increases design complexity. The authors of [51] proposed an RIS operating at 32 GHz using barium strontium titanate (BST). BST can operate with low power consumption and features a fast-tuning speed in the sub-microsecond range. Additionally, it enables continuous beam-steering, similar to varactor diodes. However, it requires a high bias voltage of several hundred volts, and large-area fabrication remains challenging. The authors of [52], [53], [54] proposed an RIS utilizing VO_2_. Previous studies have primarily focused on mm-wave bands above 30 GHz, reporting beam-steering performance of ±10° or less. VO_2_ is a material that can also be used in the THz range, offering a reasonable tuning speed in the microsecond range and enabling the implementation of low-cost RF switches. However, previous studies were limited to small-scale implementations with dimensions of 10 λ_0_ × 10 λ_0_ or less, preventing the full realization of the cost-effectiveness of VO_2_-based RF switches. We verified that these limitations can be overcome by demonstrating a low-cost, large-area 100 GHz IRS utilizing high-PCR VO_2_ and screen-printing technology.

**Table 1:** Comparison of the proposed sub-THz IRS with previous studies on reconfigurable metasurfaces.

Reference	Frequency (GHz)	FBW^a^ (%)	Size (*λ*_0_ × *λ*_0_)	Material	Switching speed	Scanning angle (°)	Cost
[48]	2	N/A	4.67 × 4.67	PIN diode	Fast	±40	High
[49]	28	N/A	6.07 × 6.07	LC	Slow	±60	Low
[50]	100	6.1	20.6 × 18.9	LC	Slow	±55	Low
[51]	32	4 (3-dB BW)	3.8 × 2.1	BST	Fast	0 to +25	High
[52]	35	13.8	10 × 10	VO_2_	Moderate	±10	Low
[53]	5, 32	N/A	N/A	VO_2_	Moderate	N/A	Low
[54]	60	15	N/A	VO_2_	Moderate	N/A	Low
This work	**100**	20	**28.6 × 17.8**	VO_2_	Moderate	**−68 to +70**	**Low**

^a^FBW (fractional bandwidth) is defined as FBW = 2(*f*_*H*_ – *f*_*L*_)/(*f*_*H*_ + *f*_*L*_), where *f*_*H*_ and *f*_*L*_ are the maximum and minimum frequency of the absorption band, respectively. Bold values highlight the key performance metrics of the proposed sub-THz IRS.

## 3 Conclusions

In summary, we demonstrated a low-cost, large-area 100 GHz IRS using screen-printable VO_2_ with a high PCR. We produced VO_2_ with a PCR of over 1,000 by controlling the synthesis temperature and annealing temperature in a hydrothermal synthesis process. The VO_2_ switch with a high PCR not only provided design freedom for the sub-THz IRS but also increased its efficiency. We verified through fabrication and measurement process that the proposed sub-THz IRS can successfully control the reflected beam angle in the sub-THz band. The proposed sub-THz IRS used VO_2_ switches instead of tuning elements (such as PIN and varactor diodes), which dramatically reduces fabrication cost and utilizes screen printing technology to enable large-area fabrication. The proposed sub-THz IRS can not only solve the NLOS issues that arise in 5G and 6G communications but also be utilized as a large-area RF electronic device to improve communication quality.

## 4 Materials and methods

### 4.1 VO_2_ µP synthesis and ink

VO_2_ µPs were synthesized using hydrothermal synthesis. Oxalic acid (12.6 g, >99 %, Sigma-Aldrich) was dissolved in deionized (DI) water (630 mL) and then V_2_O_5_ powder (2.1 g, >98 %, Sigma-Aldrich) was added. The solution was then thoroughly mixed to create a yellow slurry. The final solution was transferred into two 500 mL polypropiolactone (PPL) lined hydrothermal autoclave reactors and then held at 260 °C for 6 h. After 6 h of reaction, the reacted solution was cooled to room temperature. The resultant blue-black precipitate was centrifuged at 5,000 rpm for 3 min, then washed with DI water and ethanol using a centrifuge (DM0636, DLAB), and then dried in a vacuum oven (SH-VDO-PK-S1, SH SCIENTIFIC) at 70 °C for 1.5 h. Finally, the dried powder was annealed at 370 °C for 3 h in a vacuum oven to obtain the VO_2_ µPs. Ink formulation was produced by mixing ethyl cellulose, terpineol, and ethanol in a 1:4:0.4 weight ratio. The ethyl cellulose and terpineol were purchased from Sigma-Aldrich. The screen-printable ink was produced by mixing VO_2_ µP with the ink formulation in a 1:1 ratio.

### 4.2 Screen printing and post processing

An automatic screen printer was used for screen printing (SJ-7320A, SUNGJIN Technology), a screen mask with 150 mm × 150 mm calendered polyester screen mesh mask (400 mesh count, 22.5° mesh angle, 8 um emulsion thickness) was used for silver printing, and a screen-mask with stainless steel mesh mask (325 mesh count, 22.5° mesh angle, 8 um emulsion thickness) was used for VO_2_ printing. A total of three screen masks were used to implement the silver top pattern, ground pattern, and VO_2_ pattern, each with align marks inserted for printing alignment. The top and ground patterns were screen printed onto the quartz substrate once each using screen printable silver ink, and the VO_2_ pattern was printed over 15 cycles on the top of the quartz substrate using prepared 2.5 g VO_2_ ink. After printing the silver pattern, it was heat sintered in the oven at 130 °C for 10 min, and after printing the VO_2_, it was heat sintered on a hot plate at 90 °C for 2 h.

### 4.3 Characterization

The VO_2_ switch pattern was assessed using an FE-SEM (FE-SEM 2, Sigma300, Carl Zeiss), and the VO_2_ µP crystallinity was examined by X-ray diffraction (XRD, New D8-Advance, Bruker-AXS). The reflection coefficient of the fabricated Sub-THz IRS was measured using a Vector Network Analyzer (Agilent E8362C, Agilent) with frequency extension modules (V10VNA-T 75 GHz to 110 GHz, OML) capable of measuring up to 110 GHz. We also used rectangular horn antennas (HO10R, Custom Microwave Inc) for the transmit and receive antennas. The heat required for the phase change in VO_2_ was generated using a DC power supply (Keysight N6705C, Keysight). The motor systems comprised step motors and Arduino software.

## Supplementary Material

Supplementary Material Details
