# Supplementary material for: Low-cost large-area 100 GHz intelligent reflective surface: electrically column control of screen-printable high phase changing ratio vanadium dioxides
Source: Nanophotonics. 2025 Apr 15;14(9):1375–89. doi: 10.1515/nanoph-2025-0006 (PMC12038606; doi:10.1515/nanoph-2025-0006)
Supplement: Supplementary file 1 — Supplementary Material Details [file j_nanoph-2025-0006_suppl_001.pdf]

Supplementary material

Low-cost large-area 100 GHz intelligent reflective surface: Electrically column control of screen-printable high phase changing ratio vanadium dioxides

Eiyong Park[°]*, Junghyeon Kim*[°]*, Minjae Lee, Ratanak Phon, Mihyun Kim, Sunghoon Hong and Sungjoon Lim**

E. Park, M. Lee, S. Lim
School of Electrical and Electronic Engineering, Chung-Ang University,
84 Heukseok-Ro, Dongjak-Gu, Seoul, 06974, Republic of Korea
E-mail: sungjoon@cau.ac.kr

J. Kim, S. Lim
Department of Intelligent Semiconductor Engineering, Chung-Ang University,
84 Heukseok-Ro, Dongjak-Gu, Seoul, 06974, Republic of Korea

R. Phon
Department of Electrical and Computer Engineering, Stevens Institute of Technology,
Hoboken NJ 07030, USA

M. Kim, S. Hong
ICT Materials & Components Research Laboratory, Electronics and Telecommunications Research Institute (ETRI),
218 Gajeong-ro, Yuseong-gu, Daejeon, 34129, Republic of Korea

*Corresponding author: E-mail: sungjoon@cau.ac.kr

Supplementary data 1

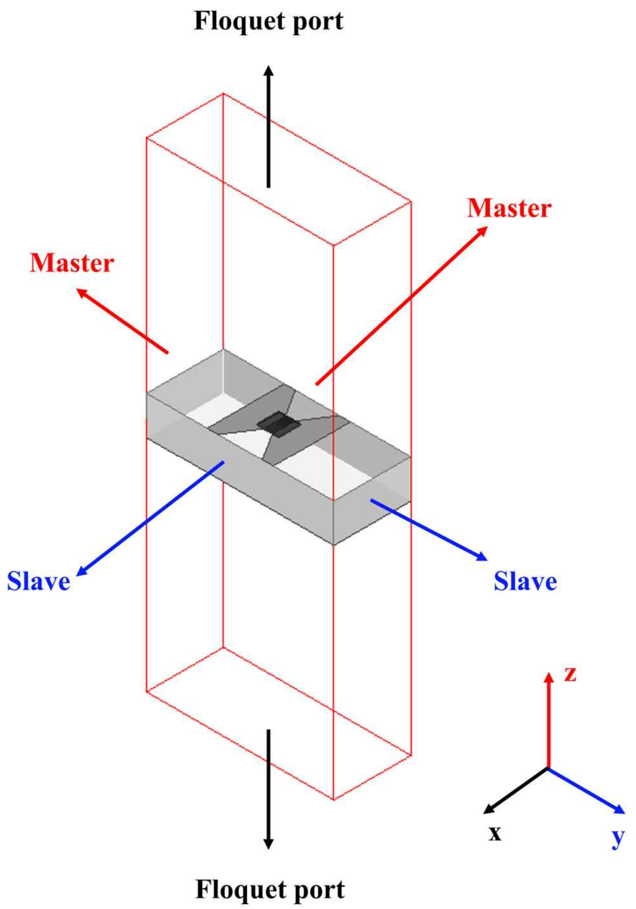

Figure S1: Unit structure simulation setup.

Supplementary data 2

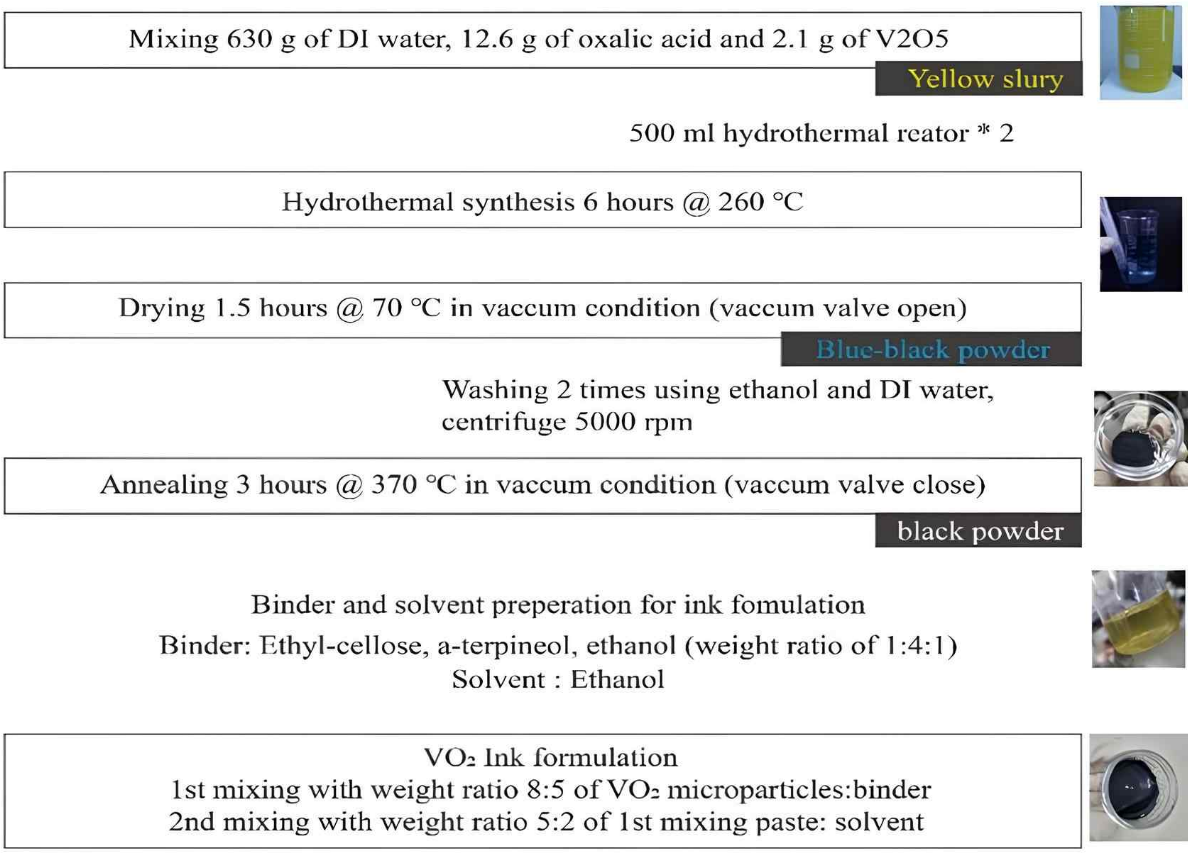

Figure S2: Process of VO_2 μP with a PCR of over 1,000 using hydrothermal synthesis and the fabricating process of ink capable of screen printing.

Supplementary data 3

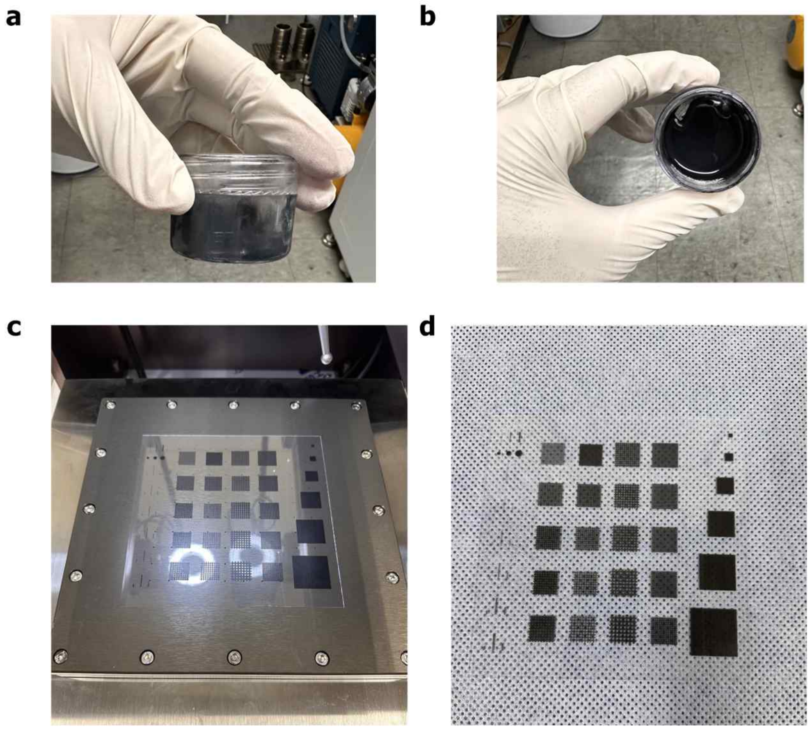

Figure S3: (a-b) Fabricated screen printable VO_2 ink. (c-d) screen printed sample using VO_2 ink.

Supplementary data 4

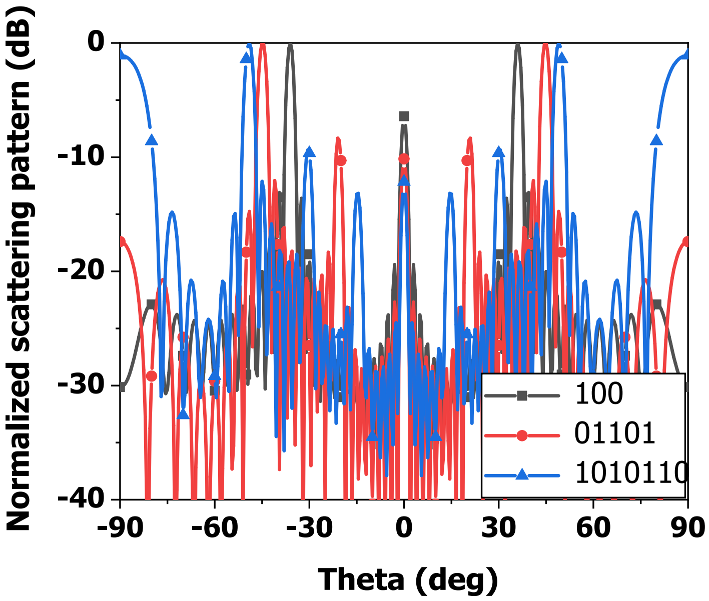

Figure S4: Calculated normalized scattering pattern results for code 100, 01101, 1010110.

Supplementary data 5

1. Screen printing 1 Cycle for silver of bottom layer

↓

2. Heat sintered in an oven at a temperature of 130 ˚C for 10 mins

↓

3. Screen printing 1 Cycle for silver of top layer

↓

4. Heat sintered in an oven at a temperature of 130 ˚C for 10 mins

↓

5. Screen printing 5 Cycles for VO_2 patterns (5 layers)

↓

6. Heat sintered on hot plate at a temperature of 90 ˚C for 30 mins

↓

7. Screen printing 5 Cycles for VO_2 patterns (10 layers)

↓

8. Heat sintered on hot plate at a temperature of 90 ˚C for 30 mins

↓

9. Screen printing 5 Cycles for VO_2 patterns (15 layers)

↓

10. Heat sintered on hot plate at a temperature of 90 ˚C for 2 hrs

Figure S5: Sub-THz IRS screen printing and sintering process.

Supplementary data 6

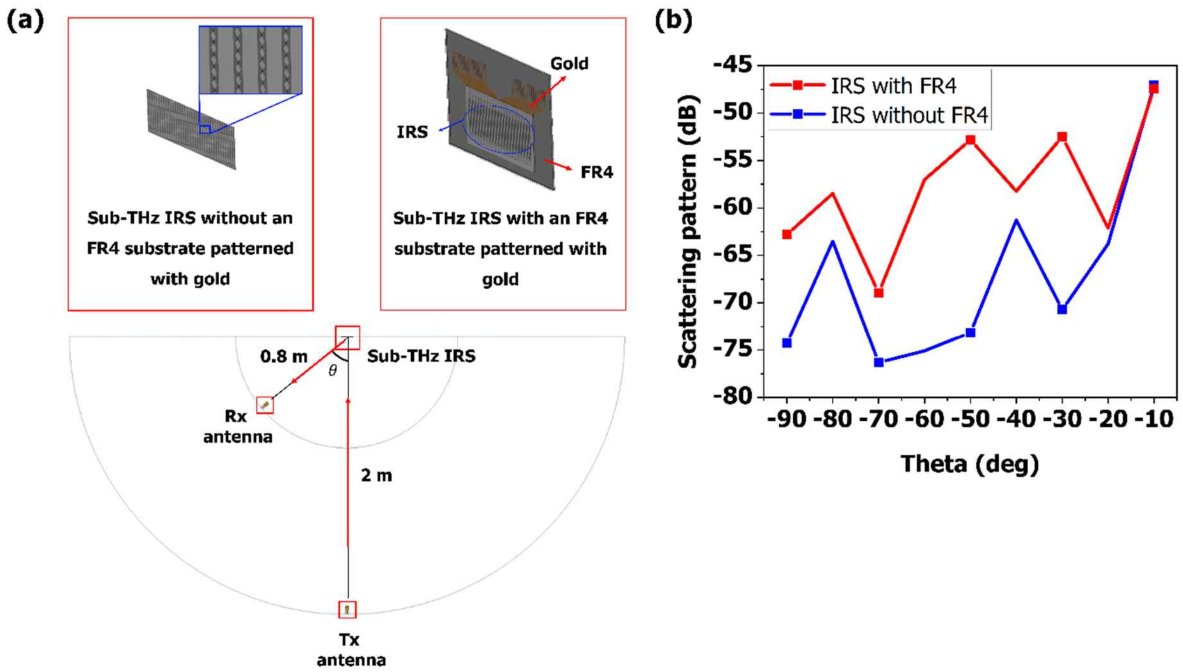

Figure S6: (a) Measurement environment implemented in Ansys HFSS. (b) Simulated scattering patterns for structures with and without gold-patterned FR4.
